# Prevalence and Predictors of Iron Deficiency at Hospital Discharge in Very-Low-Birth-Weight Infants: A Prospective Single-Center Observational Study Using RET-He and Serum Ferritin

**DOI:** 10.3390/children13060817

**Published:** 2026-06-13

**Authors:** Pacharapan Surapolchai, Phakatip Sinlapamongkolkul, Sariya Prachukthum, Tasama Pusongchai, Wallee Satayasai, Sudatip Kositamongkol

**Affiliations:** 1 Department of Pediatrics, Faculty of Medicine, Thammasat University, Pathumthani 12120, Thailand; spachara@tu.ac.th (P.S.); phakatip@tu.ac.th (P.S.); psariya@tu.ac.th (S.P.); tasamew@tu.ac.th (T.P.); swallee@tu.ac.th (W.S.); 2 Thammasat University Center of Excellence in Modern Technology and Advanced Manufacturing for Medical Innovation, Thammasat University, Pathumthani 12120, Thailand

**Keywords:** iron deficiency, very-low-birth-weight infants, reticulocyte haemoglobin equivalent, ferritin, anaemia, neonatal screening, preterm infants

## Abstract

**Highlights:**

**What are the main findings?**

- Iron deficiency was present in 39.7% of VLBW infants at hospital discharge, while anaemia occurred in only 1.5%, demonstrating that biochemical iron deficiency substantially precedes haematological change.
- Higher gestational age and lower haemoglobin at birth were independently associated with iron deficiency, likely reflecting differential exposure to iron-rich red cell transfusions across gestational age groups.

**What are the implications of the main findings?**

- Haemoglobin alone is insufficient to detect iron deficiency in VLBW infants prior to discharge; biochemical iron assessment before discharge may be clinically justified and warrants evaluation in larger multicentre studies.
- Standard iron supplementation protocols may be inadequate for more mature VLBW infants who receive fewer transfusions, warranting prospective studies to optimise supplementation strategies in this subgroup.

**Abstract:**

**Background**: Iron deficiency is common in very-low-birth-weight (VLBW) infants because of limited iron stores, rapid postnatal growth and repeated phlebotomy. Early detection is essential to prevent anaemia and neurodevelopmental impairment. This study investigated the prevalence of, and factors associated with, iron deficiency at hospital discharge using serum ferritin and the reticulocyte haemoglobin equivalent (RET-He). Methods: In this prospective cohort study, iron status was evaluated in 68 VLBW infants admitted between April 2022 and March 2024 at 36 weeks post-menstrual age (PMA) or at discharge. Iron deficiency was defined as serum ferritin below 75 ng/mL or RET-He below 28 pg. Univariable and multivariable logistic regression analyses were performed to explore clinical factors associated with iron deficiency. Iron status and anaemia were reassessed at 6–12 months of age. **Results**: At 36 weeks PMA or discharge, 39.7% of the infants were iron deficient, whereas only 1.5% were anaemic. Higher gestational age (aOR 1.81, 95% CI 1.07–3.06) and lower haemoglobin at birth (aOR 0.63, 95% CI 0.42–0.96) were independently associated with iron deficiency. Bronchopulmonary dysplasia showed a possible association (aOR 14.02, 95% CI 1.23–160.34), though this estimate should be interpreted cautiously. At 6–12 months, 18.8% of the patients had anaemia and 50% had iron deficiency, with no significant associated factors identified, likely reflecting the limited sample availability. **Conclusions:** Iron deficiency is common in VLBW infants and often precedes anaemia. Assessment of iron status beyond haemoglobin before discharge may be clinically justified to guide early supplementation, though further prospective multicentre studies are needed to confirm whether routine dual-biomarker screening is warranted.

## 1. Introduction

Iron is a vital micronutrient during early life and plays a critical role in haemoglobin (Hb) synthesis, oxygen transport, and energy metabolism. It is also essential for brain development, particularly in processes such as myelination and synaptogenesis [1]. Adequate iron endowment at birth is crucial for maintaining optimal iron status during the first 6 to 9 months of life. This endowment is influenced by maternal iron status, the timing of umbilical cord clamping, and gestational age (GA). Since the majority of foetal iron transfer occurs during the third trimester, the risk of iron deficiency is inversely related to GA [2,3,4].

Very preterm infants are vulnerable to iron deficiency due to limited iron stores at birth, frequent blood sampling, inadequate enteral iron intake, and systemic inflammation that disrupts normal iron homeostasis. Importantly, the neurodevelopmental consequences of early iron deficiency may not be fully reversible, even after iron repletion [1].

Iron status at discharge in these infants has not been extensively studied, as most previous studies have assessed iron status either during hospitalisation or after discharge. The ability to identify biochemical iron deficiency before discharge allows timely intervention before adverse outcomes develop.

Serum ferritin is widely accepted as a biomarker of iron storage. However, the use of serum ferritin has notable limitations, particularly its sensitivity to inflammation. As an acute-phase reactant, ferritin levels may be elevated in the presence of inflammatory conditions, regardless of the individual’s actual iron status. Preterm infants are frequently affected by inflammatory conditions, which may compromise the reliability of serum ferritin as an indicator of iron status in this population.

The reticulocyte haemoglobin equivalent (RET-He), a measure of Hb content in reticulocytes, offers a valuable and cost-effective alternative for assessing iron status. It can be readily obtained as part of a complete blood count without requiring additional serum samples. Importantly, RET-He is not influenced by infection or inflammation and provides a direct assessment of functional iron availability in the body. Its use has become increasingly common in paediatric populations [5].

Given that iron overload can also have detrimental effects in preterm infants, partly because of their immature antioxidant systems [6], careful monitoring of iron status is critical. Conversely, iron deficiency may have irreversible adverse effects on neurodevelopment [7]. These findings further support the need for sensitive early screening tools such as RET-He and ferritin to detect iron deficiency in VLBW infants before it progresses to overt anaemia.

At Thammasat University Hospital, RET-He is periodically assessed during hospitalisation, alongside hematocrit and reticulocyte count, to guide and adjust iron supplementation. The present study was designed as a prospective descriptive cohort report of VLBW infants cared for according to hospital standard practices. The primary aim of this study was to evaluate the iron status of VLBW infants prior to hospital discharge or at 36 weeks post-menstrual age (PMA). The secondary objective was to monitor the incidence of iron deficiency anaemia at 6–12 months of age.

## 2. Materials and Methods

### 2.1. Study Population

This was a prospective descriptive cohort study conducted in a university-based hospital that admits 50–80 very-low-birth-weight (VLBW) infants annually. Infants were eligible if they were born before 32 weeks of gestation or had a birth weight (BW) < 1500 g. Preterm infants with major congenital anomalies, those unable to tolerate full enteral feeding before 34 weeks PMA, or those with contraindications to enteral iron supplementation were excluded. Most VLBW infants receive parenteral nutrition shortly after birth. Enteral feed was gradually introduced, with an emphasis on the mother’s milk; preterm formula was used when maternal milk was unavailable. Typically, full enteral feeding was achieved within the first 10 days of life. Enteral iron supplementation was initiated during the second week of life at a dosage of 2–6 mg/kg/day and was adjusted on the basis of hematocrit, reticulocyte count, and RET-He, which were periodically monitored every 1–2 weeks. In our institution, iron supplementation was started at 3–4 mg/kg/day and adjusted according to RET-He values. When RET-He was <28 pg, the iron dose was increased by 1–2 mg/kg/day. When RET-He was between 28 and 36 pg, the existing iron dose was maintained. When RET-He was >36 pg, iron supplementation was temporarily discontinued for 2 weeks. Red cell transfusion was performed following hospital guidelines (Supplementary S1). The estimated blood loss from phlebotomy was recorded on the nursing sheet. Erythropoietin and parenteral iron were not used in our unit.

This study was approved by the Human Research Ethics Committee of Thammasat University (Medicine), Thailand (MTU-EC-PE-2-039/65). Written informed parental consent was obtained prior to enrolment. Clinical trial number not applicable.

### 2.2. Sample Size Calculation

Based on a retrospective review of medical records at Thammasat University Hospital, the estimated incidence of iron deficiency (RET-He < 28 pg) in VLBW infants at 36 weeks PMA was approximately 33%, though this estimate was derived from incomplete data and should be interpreted cautiously. Using this as a reference proportion, we estimated that the incidence of iron deficiency under a standardised iron supplementation protocol would be approximately 15%, based on anticipated clinical improvement consistent with the published literature. The sample size was calculated for a one-sample comparison of a binomial proportion using the normal approximation, testing H_0_: *p* = 0.33 versus H_1_: *p* = 0.15, with a two-sided α = 0.05 and power = 80%, a minimum of 47 infants were required for enrolment in this study. Accounting for a 25% loss to follow-up or withdrawal, the total sample size was determined to be at least 60 infants.

### 2.3. Research Methodology

Data regarding maternal and infant characteristics, the volume of blood drawn, the number and volume of red blood cell transfusions received, and the dose of iron received during hospitalisation were recorded in a data collection form. At 36 weeks PMA or before hospital discharge, whichever came first, infants underwent iron status assessments, including serum ferritin, serum iron, TIBC, RET-He, and CBC. After discharge, infants are followed by a neonatologist for general health and growth; however, some need to be followed up in their hometowns due to the universal coverage policy. At 6–12 months of age, infants were scheduled for screening for anaemia and iron deficiency. A complete blood count (CBC), including red blood cell indices, was performed immediately after blood collection via an automated cell counter (UniCel DxH 800, Beckman Coulter, Brea, CA, USA). RET-He was measured by fluorescence flow cytometry in the reticulocyte channel of the XN-1000 (Sysmex, Norderstedt, Germany). Serum ferritin, serum iron, and TIBC were analysed with a UniCel Dxl 800 (Beckman Coulter, USA).

At the 6–12-month follow-up, RET-He was not routinely measured because of local laboratory policy and was therefore excluded from the definition of iron deficiency at follow-up. We considered serum ferritin and complete blood count (CBC) sufficient to assess iron status at this age, as these parameters are widely accepted for the diagnosis of iron deficiency in infancy. Blood tests were performed only when infants were clinically stable and had no recent history of infection; therefore, we anticipated that RET-He would add little additional diagnostic value in this setting. Thalassemia screening was not included in the laboratory work-up. Micro-R and HyPO-He indices were not routinely reported from our hospital laboratory during the study period.

### 2.4. Definitions

At 36 weeks PMA: iron deficiency: serum ferritin < 75 ng/mL [8] or RET-He < 28 pg [9]; iron deficiency anaemia: iron deficiency + Hb < 8 g/dL [8] iron overload: serum ferritin > 400 ng/mL [10]). Infants were classified as iron deficient if either ferritin or RET-He was below the threshold. Discordant cases were also classified as deficient.

At 6–12 months, the iron deficiency status was as follows: iron deficiency, serum ferritin < 30 ng/mL [11]; iron deficiency anaemia, iron deficiency + Hb < 11 g/dL; and transferrin saturation, <16% [12]. All iron definition was summarized in Table 1.

Bronchopulmonary dysplasia (BPD) was diagnosed in infants who required oxygen supplementation for more than 28 days, consistent with the classification criteria applied prospectively at our institution during the study period.

The diagnosis of necrotizing enterocolitis (NEC) was made by the attending physician.

### 2.5. Statistical Analysis

The variables analysed in this study included demographic data (e.g., GA, BW, size for GA, and maternal underlying conditions), clinical data (e.g., history of anaemia, blood transfusions, treatments, and nutritional status), and laboratory data (e.g., CBC, RET-He, serum ferritin, serum iron, total iron-binding capacity (TIBC), and transferrin saturation). Descriptive statistics, including percentages (%), means with standard deviations (SDs), or medians with interquartile ranges (IQRs), depending on the data distribution, were used to summarise baseline characteristics, such as GA, BW, maternal comorbidities, the number and volume of red blood cell transfusions, and laboratory parameters, including Hb, red cell indices, and RET-He. Research hypotheses were tested via the chi-square test or Fisher’s exact test for categorical variables and the independent Student’s *t* test or Wilcoxon signed-rank test for continuous variables, as appropriate. A *p* value of <0.05 was considered statistically significant. All neonatal variables with clinical relevance to iron status were included in the multivariable model a priori, based on biological plausibility rather than univariable significance thresholds. The number of red cell transfusions was used as an indicator of transfusion exposure. The total transfusion volume was not included in the model to avoid redundancy and potential collinearity since both variables represent similar clinical information.

Iron deficiency was defined as low ferritin or low RET-He, and these markers were used exclusively as classification criteria to define iron status groups. Therefore, they were not included as explanatory variables in the regression models, in order to avoid redundancy and potential multicollinearity with the group variable. Consequently, the regression analyses should be interpreted as exploratory. Sensitivity analyses were conducted by repeating the multivariable logistic regression models using alternative iron status outcomes based on each biomarker separately. In addition to the composite iron deficiency group (low ferritin or low RET-He at 36 weeks PMA), we fitted models with low RET-He alone and low ferritin alone as dependent variables, using the same set of predictors. Results of these sensitivity analyses are presented in Supplementary S2.

For the 6-month follow-up, analyses were performed using a complete case approach, including only infants who attended follow-up and had blood sampling available. No imputation was performed for missing data. No adjustment for multiple comparisons was applied; given the exploratory nature of the analyses, all results should be interpreted accordingly.

## 3. Results

**Figure 1:**
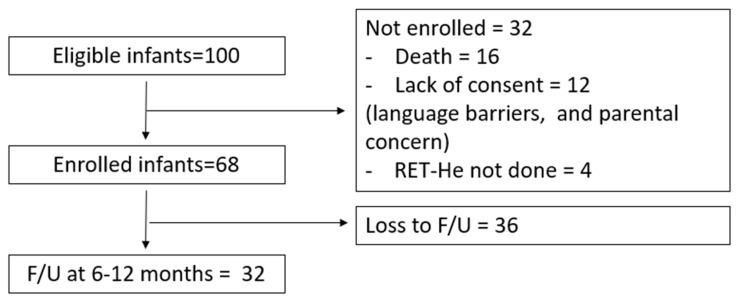
Study flow diagram.

A total of 100 infants with very-low-birth-weight (VLBW) or gestational age (GA) < 32 weeks were eligible during the study period (April 2022–March 2024). Among these, 68 infants were enrolled. Sixteen patients were excluded because of death, 12 because of language barriers or parental concerns, and 4 because of missing RET-He data. Among the enrolled infants, 32 were followed up at 6–12 months of age as shown in Figure 1.

All infants achieved full enteral feeding before 34 weeks of gestation, and none were unable to tolerate enteral iron supplementation.

Maternal and infant baseline characteristics are presented in Table 2. Most infants were VLBW. More than half of the mothers had underlying medical conditions, with some exhibiting multimorbidity. There were 50 infants (73.5%) who received blood transfusion during hospitalisation. The number of red cell transfusions during hospitalisation ranged from 0 to 10 times, with total transfused volumes ranging from 0 to 193 mL. The number of red cell transfusions was inversely correlated with gestational age (Spearman rho = −0.606, *p* < 0.001). Infants born below 30 weeks received a median of three transfusions (IQR 2–4) and 80.0% received two or more transfusions, compared with a median of 0 transfusions (IQR 0–1) and only 10.5% receiving two or more among infants born at 32 weeks or beyond (*p* < 0.001).

At 36 weeks post-menstrual age (PMA), there were no significant differences in the anthropometric measurements between infants with and without iron deficiency. The median weight was 2119 g (IQR, 1940–2357) in the iron-deficient group and 2269 g (IQR, 1972–2590) in the iron-sufficient group (*p* = 0.297). The median lengths were 44 cm (IQR, 42–45) and 44 cm (IQR, 42.5–46) in the respective groups (*p* = 0.480). Head circumference was similar between the groups, with a median of 31 cm in both groups (IQR, 30–32; *p* = 0.709).

At 36 weeks PMA, only one infant (1.47%) was anaemic, with a RET-He of 26.5 pg and a ferritin level of 127.5 ng/mL. Twenty-seven infants (39.7%) met the criteria for iron deficiency, which was defined as low RET-He or low ferritin. Table 3 summarises the haematological parameters at 36 weeks PMA.

In the univariable analysis (Table 4), no significant differences in GA, birth weight, maternal age, haemoglobin at birth, number or volume of red cell transfusions, or duration of oxygen supplementation were found between infants with and without iron deficiency.

Multivariable logistic regression identified gestational age, haemoglobin at birth, and bronchopulmonary dysplasia (BPD) as factors associated with iron deficiency at discharge. Higher gestational age was associated with increased odds of iron deficiency (aOR 1.81, 95% CI 1.07–3.06; *p* = 0.026), and lower haemoglobin at birth was similarly associated with iron deficiency (aOR 0.63, 95% CI 0.42–0.96; *p* = 0.030). The model included 11 predictors with 27 iron deficiency outcome events, yielding approximately 2.5 events per predictor. Confidence intervals were notably wide for BPD (aOR 14.03, 95% CI 1.23–160.34) and NEC (aOR 8.38, 95% CI 0.62–113.72), with the NEC estimate based on only three iron-deficient infants with this complication. The overall model showed adequate fit (pseudo R^2^ = 0.29; *p* = 0.012). Ferritin was available in 50 infants, of whom 15 had levels below 75 ng/mL. RET-He was available in all infants, with 15 having values below 28 pg. No significant correlation was observed between ferritin and RET-He (Pearson’s r = 0.098, *p* = 0.499).

### Follow-Up at 6–12 Months

A total of 32 infants completed the follow-up. Among them, 6 infants (18.8%) were diagnosed with anaemia, and 16 infants (50%) were identified as having iron deficiency. All infants received iron supplementation at a dosage of 2–4 mg/kg/day; however, compliance with iron intake was not assessed. No significant differences in growth parameters were observed between the two groups, as shown in Table 5.

According to the univariable analysis, no perinatal or neonatal variables were significantly associated with iron deficiency at 6–12 months (Table 6). Owing to the small sample size, the multivariable analysis was underpowered and not reported.

## 4. Discussion

In this prospective cohort study, 39.7% met the criteria for iron deficiency at 36 weeks PMA or discharge, whereas only 1.5% had anaemia. Gestational age, haemoglobin concentration at birth, and BPD were identified as factors associated with iron deficiency in multivariable analysis; however, these findings should be considered exploratory given the small sample size. At the 6–12-month follow-up, iron deficiency was present in 50% of the infants assessed, highlighting the persistent burden of iron deficiency beyond the neonatal period.

Iron sufficiency is essential for optimal brain development. Iron deficiency during this critical period has been associated with impaired neurodevelopmental outcomes in infants, and the effects may be irreversible, even with subsequent iron repletion [1,13]. Premature infants are at high risk of iron deficiency during early infancy. The severity of this problem is even greater in VLBW infants despite standard iron supplementation. Our findings (39.7%) fall within the previously reported range of 30–46%, though interpretation should consider population-specific factors discussed in the limitations section [14,15,16].

Most studies have reported that iron deficiency occurs after discharge during infancy; however, only a few have assessed iron status at the time of discharge. A study from the United States reported an iron deficiency incidence of 31.4% [17], whereas a study from Russia reported a rate as high as 77% before discharge [18].

The diagnosis of iron deficiency in this population remains challenging. RET-He and serum ferritin are commonly used as markers to assess iron status. RET-He reflects the functional iron available for erythropoiesis and is less influenced by inflammation, whereas ferritin reflects iron stores but may be elevated in the presence of inflammation or infection [5]. Recent studies have further confirmed the diagnostic accuracy and reliability of RET-He in paediatric populations and preterm infants, underscoring its value as a practical marker for iron deficiency screening [5,18].

In our clinical practice, RET-He is preferred over ferritin for monitoring iron status because of its lower cost, minimal blood volume requirement, and reduced susceptibility to inflammatory interference, which is consistent with recent paediatric findings [18]. This makes it a practical tool for in-hospital surveillance during iron supplementation, helping to prevent both iron deficiency and iron overload in preterm infants.

Because discordance between the two markers can occur, we classified infants as iron deficient if either RET-He or ferritin was below the defined threshold. This pragmatic approach prioritised sensitivity for identifying infants at risk of iron deficiency but may overestimate true iron deficiency prevalence. In particular, isolated low ferritin may reflect true iron store depletion, whereas isolated low RET-He may reflect transient iron-limited erythropoiesis not accompanied by reduced stores. The estimated prevalence of 39.7% should therefore be interpreted in the context of this inclusive classification strategy. Discordance between RET-He and ferritin has been previously documented in NICU populations, occurring in approximately 8% of paired measurements, most commonly as high ferritin with low RET-He in the context of inflammation or infection. In these cases, RET-He has been shown to provide a more accurate reflection of true iron status [19]. The weak correlation observed in our cohort is therefore consistent with published data and further supports the use of a dual-marker approach, prioritising sensitivity for identifying infants at risk.

Future studies should consider distinguishing between biochemical iron deficiency and iron-limited erythropoiesis to better characterise the iron status in preterm infants. A limitation of our study is that ferritin at 36 weeks PMA was not available for all infants. As a result, iron deficiency at 36 weeks PMA had to be classified based on RET-He alone in some infants, which may have led to misclassification and reduced our ability to fully characterise iron stores.

Univariable analysis did not reveal any significant associations between iron deficiency and clinical factors or infant outcomes, including GA, BW, sex, Hb at birth, the number and volume of red cell transfusions, estimated blood loss during hospitalisation, BPD, or NEC. Multivariable analysis revealed that each additional week of GA was associated with an increase in the odds ratio of iron deficiency. This finding may be explained by differential transfusion exposure. In our cohort, transfusion rates declined markedly with advancing gestational age, with the most immature infants receiving substantially more transfusions than those born at 32 weeks or beyond. As each packed red cell transfusion delivers exogenous iron, this may have partly protected the most immature infants from deficiency. These mechanisms remain exploratory and should be interpreted with caution. These findings are consistent with a Tanzanian study of 370 preterm infants, which reported higher rates of anaemia in moderate preterm infants (32–34 weeks, 51%) compared with very preterm infants (<32 weeks, 39%), after adjusting for confounders including phlebotomy exposure [20]. Higher Hb at birth had a positive effect on iron status at 36 weeks PMA. This finding supports the practice of delayed cord clamping, which facilitates placental transfusion and may improve neonatal iron stores. In our cohort, infants who received more red cell transfusions tended to have lower odds of iron deficiency, though this association did not reach statistical significance. The observed association between BPD and iron deficiency should be interpreted with considerable caution. The adjusted odds ratio of 14.03 (95% CI 1.23–160.34) is accompanied by an extremely wide confidence interval, indicating substantial imprecision rather than a reliable estimate of effect size. This instability is attributable to the small number of iron deficiency outcome events (*n* = 27) relative to the 11 predictors included in the model, raising concern about overfitting. Biologically, infants with BPD undergo frequent phlebotomy, may experience feeding difficulties, and are exposed to chronic inflammation, all of which may contribute to hepcidin-mediated iron sequestration [20]. This finding should therefore be considered hypothesis-generating only, and prospective evaluation in larger cohorts is warranted. The association between NEC and iron deficiency did not reach statistical significance (aOR 8.38, 95% CI 0.62–113.72). Critically, only three iron-deficient infants had NEC in this cohort, rendering this estimate statistically unreliable. NEC is therefore not considered a predictor of iron deficiency based on the current data and has not been carried forward to the conclusions.

Given the small number of outcome events relative to predictors (27 events, 11 predictors), all associations identified in the multivariable analysis—including gestational age, haemoglobin at birth, and BPD—should be viewed as exploratory and hypothesis-generating rather than as established independent predictors. Confirmation in adequately powered prospective studies is necessary before clinical inference can be drawn.

Only 32 infants were available for follow-up at 6–12 months of age. In our setting, preterm infants are routinely referred to their primary hospitals for long-term care after discharge, which contributed to loss to follow-up in approximately half of the cohort and resulted in a small, potentially selected sample. Infants who remained in follow-up at our centre may differ systematically from those managed elsewhere, introducing possible selection bias and limiting the generalisability of the 6–12 month of age findings. Among the 32 infants, 6 (18.8%) were found to have anaemia, and 16 (50%) had iron deficiency. RET-He was not assessed at follow-up; however, all the infants were clinically stable without any signs of infection or inflammation. No significant risk factors for anaemia during this period were identified. Given the limited sample size, multivariable analysis for factors associated with late-onset iron deficiency was not conducted. The prevalence of iron deficiency post-discharge varies widely across studies, with reported rates ranging from 32.1% to 48% [15,21,22], whereas the incidence of anaemia has been reported to be between 2.7% and 38.4% [20,23], which is consistent with the findings of our study. Interpretation of follow-up findings is limited by the small sample size, potential selection bias from loss to follow-up, and absence of data on feeding practices and supplementation adherence, which are discussed further in the limitations section. Accordingly, the observed prevalence figures at 6–12 months (18.8% anaemia and 50% iron deficiency) should be interpreted with considerable caution and may not be representative of the full cohort.

One of the key strengths of our study includes prospective data collection and reporting of iron status before hospital discharge, an aspect that has not been widely explored in the literature. The combined use of serum ferritin and RET-He enabled a comprehensive evaluation of iron status, capturing both iron stores and functional iron availability.

Several limitations of this study warrant consideration. First, the sample size was small, with only 27 iron-deficient infants, which limited the statistical power of the multivariable analysis and raises concern for overfitting. Second, loss to follow-up was approximately 53%, and infants who remained in follow-up at our centre may differ systematically from those managed elsewhere, introducing possible selection bias. Third, the follow-up interval of 6–12 months was broad, and infants assessed at different timepoints differ substantially in feeding exposure, growth velocity, and supplementation adherence, which may have introduced heterogeneity. Fourth, feeding practices including breastfeeding status, timing of complementary feeding, and use of iron-fortified formula were not systematically collected. Fifth, adherence to iron supplementation was not assessed and may represent an important determinant of late iron deficiency independent of neonatal iron status. Sixth, thalassemia screening was not performed, which is particularly relevant in our Southeast Asian population where the alpha-thalassemia trait affects an estimated 20–30% of individuals and independently causes microcytosis and reduced RET-He regardless of iron status, potentially contributing to overestimation of iron deficiency prevalence. Seventh, the proportion of infants who underwent delayed cord clamping was not documented. Eighth, BPD was classified using oxygen requirement for more than 28 days rather than the more contemporary criterion of respiratory support at 36 weeks corrected age, limiting comparability with the recent literature. Finally, the single-centre design limits generalizability of these findings. Future studies should prospectively evaluate optimal iron supplementation strategies in VLBW infants using standardised follow-up timepoints, incorporate thalassemia screening in Southeast Asian populations, and systematically document feeding practices and supplementation adherence. Multicentre studies with larger sample sizes are needed to confirm the exploratory associations identified in this study.

## 5. Conclusions

Iron deficiency is prevalent among VLBW infants and may begin before hospital discharge, often preceding anaemia. Assessment of iron status beyond haemoglobin before discharge may be clinically justified, though further prospective multicentre studies are needed to confirm whether routine dual-biomarker screening is warranted in this population. The observed association between BPD and iron deficiency should be considered hypothesis-generating and requires evaluation in larger prospective cohorts. Preventive strategies including delayed cord clamping, judicious red cell transfusion practices, optimised nutritional support, and iron supplementation tailored to high-risk infants warrant further investigation to mitigate the long-term complications of iron deficiency in this vulnerable population.

## Figures and Tables

**Table 1:** Definition and cut-off value of iron status.

Parameter	Timepoint	Definition/Threshold
Iron deficiency	36 weeks PMA	Ferritin < 75 ng/mL OR RET-He < 28 pg
Iron deficiency anaemia	36 weeks PMA	Iron deficiency + Hb < 8 g/dL
Iron overload	36 weeks PMA	Ferritin > 400 ng/mL
Iron deficiency	6–12 months	Ferritin < 30 ng/mL, Transferrin saturation < 16%
Iron deficiency anaemia	6–12 months	Iron deficiency + Hb < 11 g/dL

**Table 2:** Baseline characteristics of the study population.

Variables	Total Infants N = 68
Maternal characteristics	
Maternal age (years)	30.33 ± 6.10
Maternal diseases, *n* (%)	42 (61.76)
-Overt DM/GDM, *n* (%)	12 (17.64)
-Chronic HT/PIH, *n* (%)	15 (22.06)
-Multiple gestation, *n* (%)	18 (26.47)
Modes of delivery, *n* (%)	
-Caesarean section	39 (57.35)
Neonatal characteristics	
GA (weeks) *	30.11 ± 2.50
BW (g)	1194.17 ± 284.19
Male *n*(%)	33 (48.52)
Apgar score at 1 min **	8 (4, 9)
Apgar score at 5 min **	10 (8, 10)
NEC, *n* (%)	7 (10.29)
RDS requiring surfactant, *n* (%)	27 (39.71)
Red cell transfusions **	
-Times ***	2 (0, 4)
-Total volume (mL)	36 (0, 64)
EBL during hospitalisation (mL) **	27.5 (17.6, 43.3)
BPD, *n* (%)	43 (62.23)

Note: Data are described in *n* (%) and were analysed via Fisher’s exact test. * Data are presented as the means ± SDs and were analysed via *t* tests. ** Data are presented as the median (IQR) and were analysed via the rank-sum test. BPD, bronchopulmonary dysplasia; BW, birth weight; DM, diabetes mellitus; EBL, estimated blood loss; GA, gestational age; GDM, gestational DM; HT, hypertension; NEC, necrotizing enterocolitis; PIH, pregnancy-induced hypertension; RDS, respiratory distress syndrome *** only 50 infants received at least 1 transfusion (73.5%).

**Table 3:** Haematological parameters at 36 weeks PMA.

Variables	Iron-Deficient Infants N = 27	Iron-Sufficient Infants N = 41	*p*-Value
Hct (%) *	32.6 ± 5.2	32.0 ± 5.0	0.466
Hb (g/dL) *	10.6 ± 1.9	10.7 ± 1.8	0.992
RET-He (pg) *	27.3 ± 3.9	30.9 ± 1.5	<0.001
Ferritin (ng/mL) **	63.8 (48.5–78.3)	129.4 (104.2–203.4)	<0.001
Serum iron (µg/dL) **	90.4 (69.7–110.7)	107.2 (85.9–127.7)	0.026
TIBC (µg/dL) **	236 (187–265)	249 (219–267)	0.591
Transferrin Sat (%) *	38.6 ± 16.0	49.6 ± 17.5	0.034

* Data are presented as the means ± SDs and were analysed via *t* tests. ** Data are presented as medians (IQRs) and were analysed via the rank sum test.

**Table 4:** Univariate and multivariate analyses of factors associated with iron deficiency in infants at 36 weeks PMA.

Variables	Iron-Deficient Infants N = 27	Iron-Sufficient Infants N = 41	Univariable Analyses OR (95%CI)	Multivariable Analyses † aOR (95%CI)	*p*-Value
GA (weeks) *	30.31 ± 2.07	29.85 ± 2.64	1.08 (0.88–1.33)	1.81 (1.07–3.06)	0.026
BW (g) *	1198.96 ± 308.93	1183.83 ± 270.13	1.00 (1.00–1.00)	1.00 (0.99–1.00)	0.193
Male, *n* (%)	14 (51.85)	19 (46.34)	0.80 (0.30–2.12)	0.49 (0.11–2.10)	0.337
Maternal age (years) *	30.41 ± 4.89	29.98 ± 6.60	1.01 (0.93–1.10)		0.772
RDS required surfactant *n* (%)	7 (26)	19 (46)	0.39 (0.13–1.12)	0.22 (0.04–1.08)	0.063
Days of oxygen supplementation (days) **	44 (12, 61)	37 (17, 67)	1.0 (0.99–1.01)	1.02 (0.98–1.06)	0.381
No. of red cell transfusions **	1 (0, 3)	2 (0.5, 4)	0.89 (0.73–1.10)	0.69 (0.34–1.42)	0.315
Total volume of red cell transfusion (mL) **	40 (0, 63)	36 (8, 78.5)	1.00 (0.99–1.01)		0.427
EBL during hospitalisation (mL) **	25.4 (13.2, 36.0)	37.0 (20.9, 49.7)	0.98 (0.96–1.01)	0.96 (0.87–1.06)	0.427
Hb at Birth (g/dL) *	15.37 ± 2.24	16.06 ± 2.36	0.88 (0.71–1.09)	0.63 (0.42–0.96)	0.030
MCV at Birth (fL) *	104.71 ± 11.31	109.30 ± 9.12	0.95 (0.91–1.01)	0.98 (0.91–1.05)	0.595
BPD, *n* (%)	20 (74)	23 (56)	2.24 (0.77–6.45)	14.02 (1.23–160.3)	0.034
NEC, *n* (%)	3 (11)	4 (10)	1.13 (0.23–5.48)	8.38 (0.62–113.7)	0.110

Note: Data are described in *n* (%) and were analysed via Fisher’s exact test. * Data are presented as the means ± SDs and were analysed via *t* tests. ** Data are presented as the median (IQR) and were analysed via the rank-sum test. BPD, bronchopulmonary dysplasia; BW, birth weight; EBL, estimated blood loss; GA, gestational age; Hb, haemoglobin; MCV, mean corpuscular volume; NEC, necrotizing enterocolitis; RDS, respiratory distress syndrome. Maternal age and total volume of blood transfusion were not included in the multivariable model. Model fit: Log-likelihood = −29.22; LR χ^2^(11) = 24.12; *p* = 0.012; Pseudo R^2^ = 0.29. † Multivariable estimates should be interpreted as exploratory; the model included 27 outcome events across 11 predictors (events-per-predictor ratio ≈ 2.5), which limits the reliability of individual effect estimates.

**Table 5:** Haematological values and growth parameters of infants at 6–12 months post-natal age.

Variables	Total Infants N = 32	Iron-Deficient Infants N = 16	Iron-Sufficient Infants N = 16	*p* Value
Body weight (kg) **	7.1 (6.2, 8)	6.9 (6.4, 7.2)	7.73 (6.69, 8.63)	0.152
Length (cm) **	67 (63.6, 70)	66.5 (64.75, 68.95)	70 (66.5, 71.75)	0.50
Hct (%) **	36 (33.8, 37.9)	36.75 (34.1, 38.25)	34.8 (33.75, 36.6)	0.235
Hb (g/dL) **	11.8 (11.2, 12.8)	12.45 (11.15, 12.8)	11.3 (11.2, 12.5)	0.473
RET-He (pg) *^,^*** (*n* = 9)	25.37 ± 4.85	27.32 ± 4.69	22.93 ± 4.36	0.193
Ferritin (ng/mL) **	30.6 (20.9, 64.65)	20.9 (17.35, 25.05)	64.65 (43.7, 102.6)	<0.001
Serum iron (mcg/dL) *	69.09 ± 25.14	60.49 ± 25.38	81.38 ± 20.48	0.092
TIBC (mcg/dL) **	294 (267, 302)	300.5 (280, 321)	285 (239, 295)	0.353
Transferrin saturation (%) *	24.27 ± 12.18	19.15 ± 8.67	31.58 ± 13.29	0.033

* Data are described as the means ± SDs and were analysed via *t* tests. ** Data are presented as the median (IQR) and were analysed via the rank-sum test. *** Values are reported descriptively for the subset of infants in whom measurement was available. BW, birth weight; CI, confidence interval; Hb, haemoglobin; Hct, hematocrit; RET-He, reticulocyte haemoglobin equivalent; TIBC, total iron-binding capacity.

**Table 6:** Univariate analysis of factors affecting iron deficiency in infants at 6–12 months of postnatal age.

Factors	Iron-Deficient Infants N = 16	Iron-Sufficient Infants N = 16	Unadjusted Odds Ratio (95% CI)	*p* Value
GA (weeks) **	29.93 (28.29, 30.14)	29 (4.29, 32.14)	1.00 (0.73, 1.35)	0.982
BW (g) *	1058.5 ± 207.51	1175.13 ± 288.39	1.00 (1.00, 1.00)	0.196
Hb at birth (g/dL) *	10.99 ± 2.200	10.35 ± 1.60	0.82 (0.52, 1.31)	0.517
NEC, *n* (%)	1 (6.25)	2 (12.5)	0.47 (0.04, 5.73)	0.552
Day of oxygen supplementation (days) *	54.93 ± 24.87	51.31 ± 49.29	1.00 (0.98, 1.02)	0.792
RDS requiring surfactant, *n* (%) **	7 (43)	5 (31)	1.5 (0.36,6.69)	0.551
BPD, *n* (%)	13 (81.25)	11 (68.75)	1.97 (0.38, 10.17)	0.418
No. of red cell transfusions (times) **	2.62 (1–3.5)	2.81 (1–4)	0.97 (0.75, 1.27)	0.840
EBL during hospitalisation (mL) **	27.5 (23.9–38.3)	33.6 (16.4–47.2)	1.00 (0.96, 1.04)	0.946
Ferritin at 36 weeks (ng/mL)	76.55 (66.65–101.3)	108.5 (74.1–187.7)	1.01 (0.99, 1.02)	0.230
RET-He at 36 weeks (pg) **	29.35 (27.49–31.20)	29.07 (26.77–31.37)	1.02 (0.84, 1.23)	0.837

Note: Data are described in *n* (%) and were analysed via Fisher’s exact test. * Data are presented as the means ± SDs and were analysed via *t* tests. ** Data are presented as the median (IQR) and were analysed via the rank-sum test. BPD, bronchopulmonary dysplasia; BW, birth weight; CI, confidence interval; EBL, estimated blood loss; GA, gestational age; Hb, haemoglobin; NEC, necrotizing enterocolitis; RDS, respiratory distress syndrome; RET-He, reticulocyte haemoglobin equivalent.

## Data Availability

The data presented in this study are available on request from the corresponding author due to privacy and ethical reasons.

## Supplementary Materials

The following supporting information can be downloaded at: https://www.mdpi.com/article/10.3390/children13060817/s1, Supplementary S1: Transfusion guideline of our institution. Supplementary S2: Sensitivity analysis of low RET-He, low ferritin, and combined iron deficiency criteria at 36 weeks PMA, Supplementary S3: Institutional iron supplementary protocol.

## Abbreviations

The following abbreviations are used in this manuscript:

BW	birth weight
BPD	bronchopulmonary dysplasia
CBC	complete blood count
GA	gestational age
Hb	haemoglobin
IQR	interquartile range
NEC	necrotizing enterocolitis
PMA	post-menstrual age
RET-He	reticulocyte haemoglobin equivalent
SD	standard deviation
TIBC	total iron-binding capacity
VLBW	Very-low-birth-weight
