# Supplementary material for: Prevalence and Predictors of Iron Deficiency at Hospital Discharge in Very-Low-Birth-Weight Infants: A Prospective Single-Center Observational Study Using RET-He and Serum Ferritin
Source: Children (Basel). 2026 Jun 13;13(6):817. doi: 10.3390/children13060817 (PMC13297462; doi:10.3390/children13060817)

Supplementary S1: Blood Transfusion Guideline in Preterm Infants

Postnatal Week	Hemoglobin (g/dL)	
	No Respiratory Support	Respiratory Support (e.g., Supplemental Oxygen, HFNC, CPAP, PEEP)
Week 1	10–12	11–13
Week 2	8.5–11	10–12.5
≥ Week 3	7–10	8.5–11

Additional Considerations for Transfusion:

- The decision to transfuse should consider not only hemoglobin levels but also clinical factors such as:
  - History of blood loss (e.g., hemolysis, frequent blood sampling, surgery)
  - Nutritional status
  - Severity of illness
  - Hemoglobin levels obtained from arterial or venous samples may be

lower than from capillary sampling

Calculation of Blood Volume for Transfusion in Neonates:

- Always specify the volume of blood in milliliters (ml) and the duration of transfusion.
- Typical transfusion volume is 10–20 ml/kg. A dose of 20 ml/kg may be given in cases of severe anemia with evidence or risk of ongoing blood loss.
- Consider administering furosemide before transfusion in infants at risk of fluid overload, such as those with:
  - Fluid retention
  - Chronic lung disease
  - Congestive heart failure
  - Acute kidney injury

Supplementary S2: Sensitivity analysis of low RET-He, Low Ferritin and combine for diagnosis of iron deficiency

Predictor	Model 1 – Low RET-He at 36 wks OR (95% CI); p	Model 2 – Low ferritin at 36 wks OR (95% CI); p	Model 3 – Iron deficiency group at 36 wks OR (95% CI); p
Gestational age (per week)	2.11 (1.07–4.14); p=0.030	0.99 (0.53–1.86); p=0.983	1.81 (1.07–3.06); p=0.026
Birth weight (per g)	1.00 (0.99–1.00); p=0.279	1.00 (0.99–1.00); p=0.868	1.00 (0.99–1.00); p=0.193
Male sex	2.35 (0.32–17.16); p=0.399	0.05 (0.00–0.68); p=0.024	0.49 (0.11–2.10); p=0.337
Surfactant use	0.26 (0.02–3.50); p=0.312	0.85 (0.12–5.84); p=0.869	0.22 (0.04–1.08); p=0.063
Days of oxygen supplementation	1.02 (0.97–1.08); p=0.386	1.03 (0.97–1.10); p=0.282	1.02 (0.98–1.06); p=0.381
Number of red cell transfusions	1.38 (0.53–3.62); p=0.508	0.27 (0.06–1.28); p=0.099	0.69 (0.34–1.42); p=0.315
Estimated blood loss during hospitalization	0.93 (0.82–1.07); p=0.324	0.89 (0.72–1.09); p=0.255	0.96 (0.87–1.06); p=0.427
Hemoglobin at birth	0.69 (0.41–1.17); p=0.165	0.68 (0.40–1.15); p=0.151	0.63 (0.42–0.96); p=0.030
MCV at birth	0.90 (0.81–1.01); p=0.077	1.09 (0.96–1.25); p=0.193	0.98 (0.91–1.05); p=0.595
BPD	8.34 (0.47–149.38); p=0.150	1.57 (0.11–21.68); p=0.737	14.03 (1.23–160.34); p=0.034

Predictor	Model 1 – Low RET-He at 36 wks OR (95% CI); p	Model 2 – Low ferritin at 36 wks OR (95% CI); p	Model 3 – Iron deficiency group at 36 wks<br><br>OR (95% CI); p
NEC	6.37 (0.21–194.96); p=0.289	95.60 (0.39–23603.36); p=0.105	8.38 (0.62–113.72); p=0.110

Model 1: n=61, pseudo R^2=0.4073. Model 2: n=47, pseudo R^2=0.3673.
Model 3: n=61, pseudo R^2=0.2921.

Supplementary S3: Institutional iron supplementary protocol

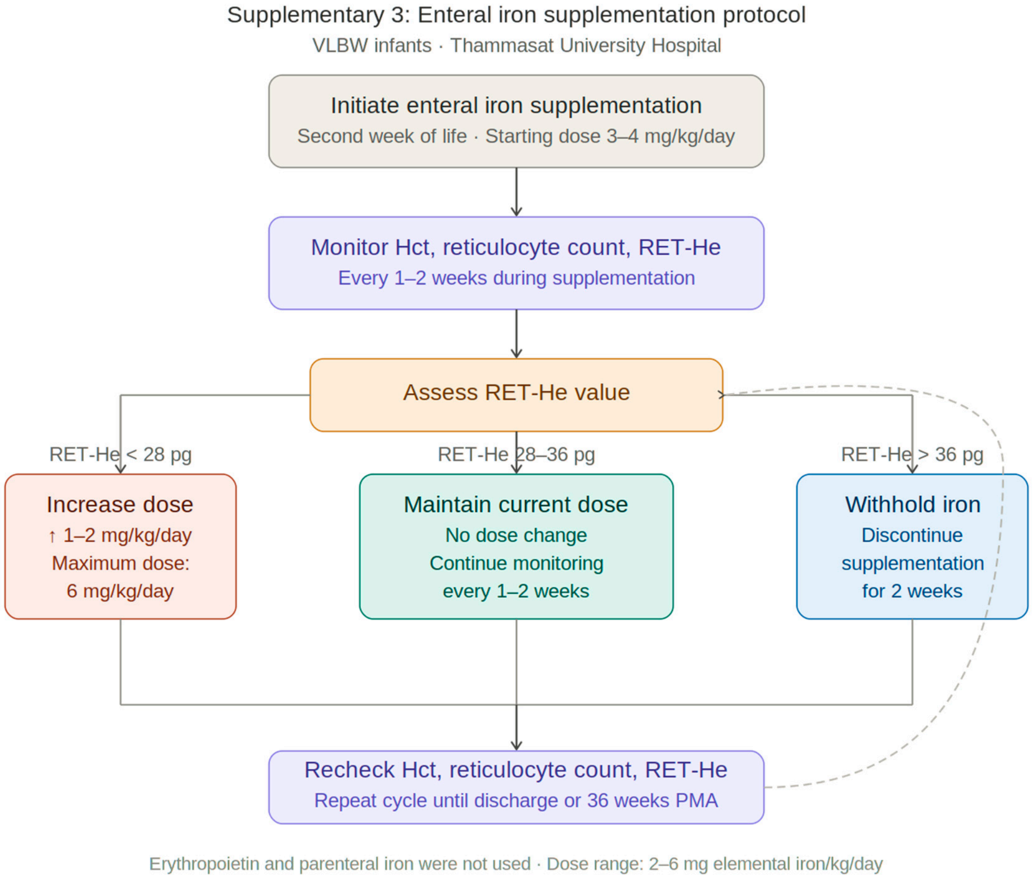

Supplement: Supplementary file 1 [file children-13-00817-s001.zip › children-4338282-supplementary.pdf]
